# Refined discrete and empirical horizontal gradients in VLBI analysis

**DOI:** 10.1007/s00190-018-1127-1

**Published:** 2018-02-20

**Authors:** Daniel Landskron, Johannes Böhm

**Affiliations:** Technische Universität Wien, Vienna, Austria

**Keywords:** VLBI, GNSS, Troposphere, Horizontal gradients

## Abstract

Missing or incorrect consideration of azimuthal asymmetry of troposphere delays is a considerable error source in space geodetic techniques such as Global Navigation Satellite Systems (GNSS) or Very Long Baseline Interferometry (VLBI). So-called horizontal troposphere gradients are generally utilized for modeling such azimuthal variations and are particularly required for observations at low elevation angles. Apart from estimating the gradients within the data analysis, which has become common practice in space geodetic techniques, there is also the possibility to determine the gradients beforehand from different data sources than the actual observations. Using ray-tracing through Numerical Weather Models (NWMs), we determined discrete gradient values referred to as GRAD for VLBI observations, based on the standard gradient model by Chen and Herring (J Geophys Res 102(B9):20489–20502, 1997. 10.1029/97JB01739) and also for new, higher-order gradient models. These gradients are produced on the same data basis as the Vienna Mapping Functions 3 (VMF3) (Landskron and Böhm in J Geod, 2017. 10.1007/s00190-017-1066-2), so they can also be regarded as the VMF3 gradients as they are fully consistent with each other. From VLBI analyses of the Vienna VLBI and Satellite Software (VieVS), it becomes evident that baseline length repeatabilities (BLRs) are improved on average by 5% when using a priori gradients GRAD instead of estimating the gradients. The reason for this improvement is that the gradient estimation yields poor results for VLBI sessions with a small number of observations, while the GRAD a priori gradients are unaffected from this. We also developed a new empirical gradient model applicable for any time and location on Earth, which is included in the Global Pressure and Temperature 3 (GPT3) model. Although being able to describe only the systematic component of azimuthal asymmetry and no short-term variations at all, even these empirical a priori gradients slightly reduce (improve) the BLRs with respect to the estimation of gradients. In general, this paper addresses that a priori horizontal gradients are actually more important for VLBI analysis than previously assumed, as particularly the discrete model GRAD as well as the empirical model GPT3 are indeed able to refine and improve the results.

## Introduction

During their passage through the neutral atmosphere, radio waves are delayed and bent as a result of interaction with dry gases and water particles. As there is no chance to directly measure the amount of delay with sufficient accuracy, the delays are usually modeled instead. While the elevation angle-dependent part of the delay is taken into account by the use of mapping functions, the delay also depends significantly on the azimuth of the observation. The ellipsoidal shape of the troposphere as well as the temporally and spatially varying refractivity of the air cause the delays to vary significantly for different observed azimuth angles. In most cases, this effect is considered through horizontal troposphere gradients multiplied with sine and cosine functions, intended to model symmetric variations over the azimuth range. Consideration of these gradients is particularly important for the realization of celestial reference frames (CRFs) (MacMillan and Ma 1997) and terrestrial reference frames (TRFs) (Böhm and Schuh 2007; Mayer et al. 2017). In the analysis of space geodetic techniques such as Global Navigation Satellite Systems (GNSS) and Very Long Baseline Interferometry (VLBI), it has become common practice to estimate gradients on the basis of a very high number of observations. In GNSS, these gradient values are determined and published for instance by the International GNSS Service (IGS), while in VLBI they are important output quantities of analysis software. However, horizontal gradients can also be determined from sources other than the actual observations. Ray-tracing through numerical weather models (NWMs) has proven to be well suited for deriving troposphere delays and hence has become the basis for the most accurate mapping functions currently available. In these NWMs, the lower atmosphere is discretized to a temporally varying three-dimensional grid, where the ray-tracing beams then propagate through. Following the Eikonal equation, the ray-tracing beams are delayed and bent, simulating the real travel path as well as possible. As the NWMs are available globally, ray-traced delays can be produced for any point on Earth. The ray-tracing software developed by Hofmeister and Böhm (2017) as part of the Vienna VLBI and Satellite software (VieVS) (Böhm et al. 2017) can not only be used for the derivation of highly accurate mapping functions [see Landskron and Böhm (2017)], but provides the basis for the determination of horizontal troposphere gradients through 2D ray-tracing at several azimuth angles, too. Depending on the underlying gradient model, the gradients can be realized at all NWM epochs for any site on Earth.

Yet, only minor importance was attached to a priori gradients in VLBI, underlined by the negligible number of existing models and realizations. The Linear Horizontal Gradients (LHG) by Böhm and Schuh (2007) represent the only existing discrete a priori gradient model for VLBI. Calculated directly from NWMs (without ray-tracing), these gradients are provided for all VLBI stations at each NWM epoch, intended for a priori use in VLBI analysis. The term "discrete" in this context means that the gradients are determined discretely for certain locations and times, generally from up-to-date information from ray-tracing through NWMs. In contrast, empirical models rely on experience values from climatology instead. Hereof, two models need to be mentioned: the DAO model from the Data Assimilation Office (MacMillan and Ma 1997) which has been determined by vertical integration over horizontal refractivity gradients, as well as the APG model (Böhm et al. 2013), which first applied the technique of ray-tracing through monthly mean pressure level re-analysis data of the ECMWF. The gradients from these models can then be applied in VLBI analyses as a priori values.

Section 2 first gives a basic understanding of azimuthal asymmetry in troposphere delay modeling. In Sect. 3 the generation of new gradient models is described, whose performance is then assessed in Sect. 4, leading to the conclusions in Sect. 5.

## Fundamentals of horizontal gradients

The modeling of troposphere delays without consideration of azimuthal variations is commonly handled with Eq. (1) [e.g., Nilsson et al. (2013)]:

1 $$\begin{aligned} \varDelta L_0(\varepsilon ) = \varDelta L_\mathrm{h}^z \cdot mf_\mathrm{h}(\varepsilon ) + \varDelta L_\mathrm{w}^z \cdot mf_\mathrm{w}(\varepsilon ) \end{aligned}$$

The delay is split into a hydrostatic and a wet component, where $\varDelta L_\mathrm{h}^z$ and $\varDelta L_\mathrm{w}^z$ denote the delays in zenith direction and $mf_\mathrm{h}(\varepsilon )$ and $mf_\mathrm{w}(\varepsilon )$ are the mapping functions accounting for the hydrostatic and the wet part as a function of the elevation angle $\varepsilon$.

In order to model variations in the delays not only depending on the elevation angle but also on the azimuth angle of the observation, a further term must be added to Eq. (1). Gardner (1977) was the first to introduce formulae to compensate for the effect of azimuthal asymmetry. Twenty years later, Chen and Herring (1997) proposed the following formula for the modeling of azimuthal asymmetry:

2 $$\begin{aligned} \varDelta L(\alpha ,\varepsilon )= & {} \underbrace{\varDelta L_0(\varepsilon )}_{\text {isotropic part}} \nonumber \\&+ \underbrace{mf_\mathrm{g}(\varepsilon ) \cdot \left[ G_\mathrm{n} \cdot \hbox {cos}(\alpha )+G_\mathrm{e} \cdot \hbox {sin}(\alpha )\right] }_{\text {anisotropic part}} \end{aligned}$$

which is of common usage in GNSS as well as VLBI analysis down to the present day. The defining variables are the north gradient $G_\mathrm{n}$ and the east gradient $G_\mathrm{e}$ which determine the variation of the delays with varying azimuth, based on the idea of a tilting of the atmosphere (Herring 1992). The term $mf_\mathrm{g}(\varepsilon )$ denotes the gradient mapping function, which models the higher refractivity at smaller elevation angles due to the longer signal path. The representation by Chen and Herring (1997), assuming an exponential decay of the horizontal gradient with increasing height, has prevailed:

3 $$\begin{aligned} mf_\mathrm{g}(\varepsilon ) = \frac{1}{\hbox {sin}(\varepsilon ) \cdot \hbox {tan}(\varepsilon )+C} \end{aligned}$$

The gradient mapping function coefficient *C* can be written as:

4 $$\begin{aligned} C = \frac{3H}{R_\mathrm{e}} \end{aligned}$$

The scale height *H* is the height of the neutral atmosphere assuming constant density with height and conservation of the total mass (Nilsson et al. 2013). Assuming a hydrostatic scale height $H_{\mathrm{h}}$ of 6.5 km and a wet scale height $H_{\mathrm{w}}$ of 1.5 km, Chen and Herring (1997) get values of $C_{\mathrm{h}} = 0.0031$ and $C_{\mathrm{w}} = 0.0007$ for the gradient mapping function coefficient, $R_\mathrm{e}$ being the Earth radius. For modeling total gradients, the factor $C = 0.0032$ is recommended (Herring 1992).

Azimuthal asymmetry originates from a number of effects:

- The rotation of the Earth and its resulting centrifugal force not only turn the Earth into an ellipsoid, but act on the atmosphere as well. Consequently, the troposphere is thicker at the equator than at the poles by some kilometers. This effect, which is also referred to as the atmospheric bulge, systematically acts on electromagnetic signals traveling through the troposphere, more precisely on the hydrostatic part; the longer a signal’s path, the larger its delay. At the equator, the systematic effect is fairly equal for signals from the north and from the south. At the poles, it is equal for all cardinal directions. Given the site of the VLBI station WETTZELL in southern Germany at a latitude of 49$^{\circ }$, for instance, signals arriving from the north are less delayed than signals from the south.
- Space geodetic techniques as well as ray-tracing through NWMs usually refer to the reference ellipsoid. However, the real shape of the Earth is much more complex, being referred to as the geoid. Deflections of the vertical (DOV) are the angles between the plumb line and lines perpendicular to the reference ellipsoid at certain locations. In reverse, these DOV can also be visualized as horizontal gradients. They are particularly distinct at plate boundaries or near major mountain ranges.
- Higher temperatures lead to higher convection which lifts the tropopause upwards, which is why the thickness of the troposphere is generally lower in cold conditions and higher in warm conditions (Geerts and Linacre 1997). As a consequence, the tropopause over the poles is up to 2 km higher in summer than in winter.
- The refractivity along the signal path, which mainly depends on temperature, pressure, humidity, $CO_2$ composition and density (Jones 1981), is highly variable both temporally and spatially. As a result, signals reaching a station from different cardinal directions experience different delays, which is considered as a random effect.

## Development of new horizontal gradients

This section presents the determination of new north gradients $G_\mathrm{n}$ and east gradients $G_\mathrm{e}$ for the gradient formula by Chen and Herring (1997) as well as for new, higher-order gradient formulae. The main goals for the new gradients are to outperform existing models in VLBI analysis, as well as to improve the baseline length repeatability (BLR) of VLBI analysis w.r.t. estimating the gradients. The basis for the determination are ray-traced delays from the VieVS ray-tracer applying the 2D piece-wise linear approach (Hobiger et al. 2008). Unlike 1D ray-tracing, in the 2D approach lateral changes in refractivity are also considered.

### Determination of discrete horizontal gradients for VLBI

The bulk of this paper is devoted to the determination of new realizations of discrete horizontal gradients based on the standard gradient formula Eq. (2), referred to as GRAD-1, which are then applied in VLBI analysis as well as in delay comparisons. In addition, two extended gradient formulae including higher-order terms are introduced:

5 $$\begin{aligned} \varDelta L(\alpha ,\varepsilon )= & {} \varDelta L_0(\varepsilon ) + mf_\mathrm{g}(\varepsilon ) \cdot \nonumber \\&[G_\mathrm{n} \cdot cos(\alpha )+G_\mathrm{e} \cdot \hbox {sin}(\alpha ) \nonumber \\&+\,G_{\mathrm{n}_2} \cdot \hbox {cos}(2\alpha )+\,G_{\mathrm{e}_2} \cdot \hbox {sin}(2\alpha )] \end{aligned}$$

6 $$\begin{aligned} \varDelta L(\alpha ,\varepsilon )= & {} \varDelta L_0(\varepsilon ) + mf_\mathrm{g}(\varepsilon ) \cdot \nonumber \\&[G_\mathrm{n} \cdot \hbox {cos}(\alpha )+G_\mathrm{e} \cdot \hbox {sin}(\alpha ) \nonumber \\&+\,G_{\mathrm{n}_2} \cdot \hbox {cos}(2\alpha )+\,G_{\mathrm{e}_2} \cdot \hbox {sin}(2\alpha ) \nonumber \\&+\,G_{\mathrm{n}_3} \cdot \hbox {cos}(3\alpha )+\,G_{\mathrm{e}_3} \cdot \hbox {sin}(3\alpha )] \end{aligned}$$

The term $G_\mathrm{n} \cdot \hbox {cos}(\alpha )$ determines the azimuthal asymmetry in north–south direction, whereas $G_\mathrm{e} \cdot \hbox {sin}(\alpha )$ determines the azimuthal asymmetry in east–west direction. Thus, one positive and one negative extremum in the asymmetric delay residuals can be modeled. Due to the simple sinusoidal structure of the model, a shortcoming is that a maximum in any azimuthal direction is always accompanied by a respective minimum of opposite sign in an angular distance of 180$^{\circ }$. This describes systematic effects like the atmospheric bulge very well, but random effects such as weather fronts or variable atmosphere heights due to local temperature differences set limits in such a way that the consequent extremum does not have a counterpart in the opposite direction. The higher-order gradient variables are intended to model the azimuthal delay variation more closely. The gradients from the standard gradient formula Eq. (2) are henceforth referred to as GRAD-1, those from Eq. (5) as GRAD-2 and those from Eq. (6) as GRAD-3. The term GRAD is used as an umbrella term for all of them.

The gradients $G_\mathrm{n}$, $G_\mathrm{e}$, $G_{\mathrm{n}_2}$, $G_{\mathrm{e}_2}$, $G_{\mathrm{n}_3}$ and $G_{\mathrm{e}_3}$ have to be determined in least-squares adjustments. The ray-traced slant delays $\varDelta L(\alpha ,\varepsilon )$ come from ray-tracing following the specifications listed in Table 1. The elevation angles were picked in such a way as to cover the whole elevation range, while the number of azimuth angles had to be large enough to ensure a sufficient over-determination for the subsequent least-squares adjustment.

**Table 1:** Properties of the ray-traced delays that were generated using the VieVS ray-tracer from 1999 to 2014

Parameter	Specification
Ray-tracing software	VieVS ray-tracer (Hofmeister and Böhm 2017)
Ray-tracing method	2D piece-wise linear (Hobiger et al. 2008)
NWM	ECMWF ERA-Interim pressure level data + ECMWF operational data
Horizontal resolution of the NWM	$1^{\circ }\times 1^{\circ }$
Vertical coverage	25 pressure levels
Horizontal coverage	33 VLBI stations
Temporal resolution	6-hourly at 00:00, 06:00, 12:00 and 18:00 UTC each day from 1999 through 2014 ($=$ 23,376 epochs)
Outgoing elevation angles per point	7 ($3^{\circ }$, $5^{\circ }$, $7^{\circ }$, $10^{\circ }$, $15^{\circ }$, $30^{\circ }$ and $70^{\circ }$)
Azimuth angles per point	16 ($0^{\circ }$:22.5$^{\circ }$:337.5$^{\circ }$)

First, for each elevation angle and station the slant delays of all 16 azimuths are averaged in order to simulate azimuthally isotropic signals $\varDelta L_0(\varepsilon )$. Through subtracting $\varDelta L_0(\varepsilon )$ from the $\varDelta L(\alpha ,\varepsilon )$, only the asymmetric parts of the delays $\varDelta L_{\mathrm{res}}(\alpha ,\varepsilon )$ at each azimuth remain. This changes Eq. (2) to:

7 $$\begin{aligned} \varDelta L_{\mathrm{res}}(\alpha ,\varepsilon ) = mf_\mathrm{g}(\varepsilon ) \cdot \left[ G_\mathrm{n} \cdot \hbox {cos}(\alpha )+G_\mathrm{e} \cdot \hbox {sin}(\alpha )\right] \end{aligned}$$

Equations (5) and (6) are altered likewise. As the left side of Eq. (7) is known from ray-tracing, the unknowns $G_\mathrm{n}$ and $G_\mathrm{e}$ can be determined through an unweighted least-squares adjustment using partial derivatives. In fact, this is done each for the hydrostatic and the wet part, resulting in gradients $G_{\mathrm{n_h}}$, $G_{\mathrm{e_h}}$, $G_{\mathrm{n_w}}$ and $G_{\mathrm{e_w}}$.

**Fig. 1:**
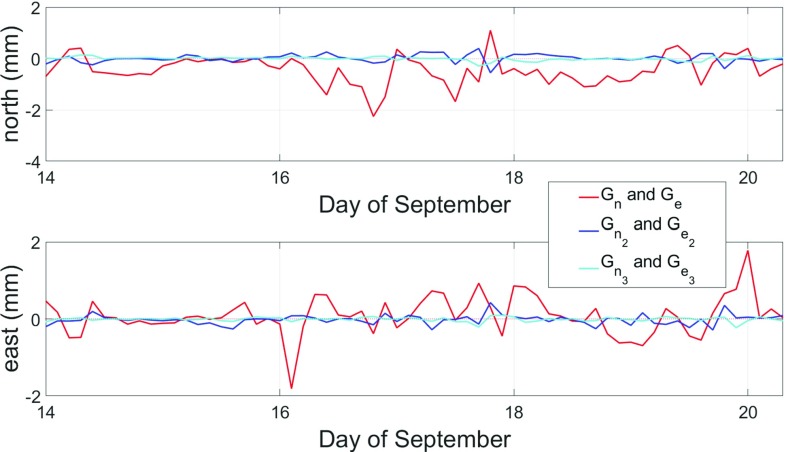
Comparison of the various gradients for station WESTFORD (Westford, Massachusetts, USA) in mid-September 2011

Figure 1 indicates that $G_\mathrm{n}$ and $G_\mathrm{e}$ are considerably larger in size than $G_{\mathrm{n}_2}$, $G_{\mathrm{e}_2}$, which in turn are larger than $G_{\mathrm{n}_3}$ and $G_{\mathrm{e}_3}$.

**Fig. 2:**
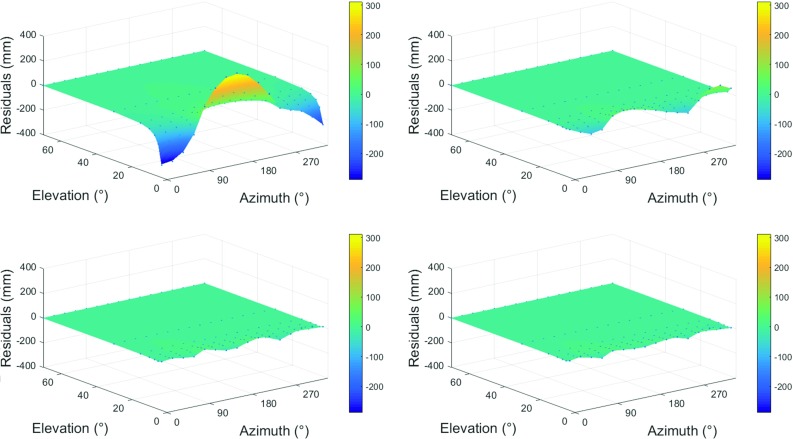
Comparison of the residuals in slant total delay for station WESTFORD (Westford, Massachusetts, USA) for the epoch September 26, 2011, 18:00 GMT [from Landskron et al. (2015a)]. Top left: residuals after subtraction of a mean over the 16 constantly distributed azimuths without applying any gradient model; the yellow and blue amplitudes mainly show the presence of the atmospheric bulge, the influence of which is highest for low-elevation observations. Top right: residuals after applying GRAD-1; thus, the bulk of azimuthal asymmetry is explained; however, small amplitudes between the cardinal points remain. Bottom left: applying GRAD-2 further lowers the residuals considerably, also the amplitudes between the cardinal points almost vanish. Bottom right: after applying GRAD-3, the residuals hardly change compared to GRAD-2

The capability of GRAD-1, GRAD-2 and GRAD-3 to describe the azimuthal asymmetry can be assessed by determination of the residuals between the modeled delays and the ray-traced delays. Figure 2 shows this exemplarily for VLBI station WESTFORD on September 26, 2011, 18:00 GMT. Averaging the residuals in the slant total delays over all 14 stations and all 15 days of the CONT11 campaign shows a decrease by 69% when using the standard gradient formula Eq. (2) (GRAD-1), by 78% when using the second gradient formula Eq. (5) (GRAD-2) and by 81% when using the third gradient formula Eq. (6) (GRAD-3) compared to non-consideration of gradients. In other words, two-thirds of the azimuthal asymmetry can be described by the standard gradient formula and even more when using extended gradient formulae. This is a first clear indicator that the extended gradient formulae are indeed capable of describing azimuthal asymmetry more precisely.

### Determination of an empirical gradient grid

Apart from the discrete horizontal gradients GRAD, there is also a new empirical gradient grid determined as part of the Global Pressure and Temperature 3 (GPT3) model, providing empirical values for $G_{\mathrm{n_h}}$, $G_{\mathrm{e_h}}$, $G_{\mathrm{n_w}}$ and $G_{\mathrm{e_w}}$. Empirical gradient models are needed particularly for observations in the early years of VLBI up to about 1990 (Spicakova et al. 2011), when only few stations were observing a small number of sources, resulting in a non-uniform sky coverage that limits the ability of estimating the gradients in a least-squares adjustment (Heinkelmann and Tesmer 2013). However, empirical gradients may also be important for recent data, for instance for the purpose of deriving terrestrial reference frames (TRFs) from VLBI or for high latitude sites in general where the effect of the atmospheric bulge is most distinct (Böhm et al. 2011).

Currently, only the empirical gradient models APG and DAO are of importance. APG is globally applicable based on a spherical harmonics expansion up to degree and order nine, whereas DAO is only available for a selected list of 174 VLBI stations (as of 2016/05), with new ones being added regularly. Both models provide only total gradients and no separated hydrostatic and wet parts. For VLBI analysis, Böhm et al. (2011) recommend using DAO rather than APG.

For the determination of a new empirical gradient grid, discrete horizontal gradients $G_\mathrm{n}$ and $G_\mathrm{e}$ (GRAD-1) were calculated first on two global grids following the specifications listed in Table 2. The extended gradient variables GRAD-2 and GRAD-3 are not considered here since their influence is too small for empirical modeling. The next step is to deduce empirical approximations from these discrete gradients, namely mean values of both hydrostatic and wet $G_\mathrm{n}$ and $G_\mathrm{e}$ for each grid point plus their annual and semiannual amplitudes. The following seasonal fit formula is applied (Lagler et al. 2013; Böhm et al. 2015), providing both a spatial and a temporal variation, exemplified here for the hydrostatic north gradient $G_{\mathrm{n_h}}$:

8 $$\begin{aligned} G_{\mathrm{n_h}}= & {} A_0 + A_1\cdot \cos \left( \frac{doy}{365.25}2\pi \right) + B_1\cdot \sin \left( \frac{doy}{365.25}2\pi \right) \nonumber \\&+ \,A_2\cdot \cos \left( \frac{doy}{365.25}4\pi \right) + \,B_2\cdot \sin \left( \frac{doy}{365.25}4\pi \right) \nonumber \\ \end{aligned}$$

**Table 2:** Properties of the grid-wise ray-traced delays that were generated for the derivation of the empirical gradient grids GPT3 ($1^{\circ }\times 1^{\circ }$) and GPT3 ($5^{\circ }\times 5^{\circ }$)

Parameter	Specification
Ray-tracing software	VieVS ray-tracer (Hofmeister and Böhm 2017)
Ray-tracing method	2D piece-wise linear (Hobiger et al. 2008)
NWM	ECMWF ERA-Interim pressure level data
Horizontal resolution of the NWM	$1^{\circ }\times 1^{\circ }$
Horizontal coverage	(1) global grid with resolution $5^{\circ }\times 5^{\circ }$ (lat: [$87.5^{\circ }$, $-87.5^{\circ }$], lon: [$2.5^{\circ }$, $357.5^{\circ }$]), resulting in 2592 grid points and (2) global grid with resolution $1^{\circ }\times 1^{\circ }$ (lat: [$89.5^{\circ }$, $-89.5^{\circ }$], lon: [$0.5^{\circ }$, $359.5^{\circ }$]) resulting in 64,800 grid points
Vertical coverage	25 pressure levels
Temporal resolution	Mean values for every month from 2001 through 2010 ($=$ 120 epochs)
Outgoing elevation angles per point	4 ($3.3^{\circ }$, $5^{\circ }$, $15^{\circ }$ and $30^{\circ }$) for $5^{\circ }\times 5^{\circ }$ grid and 1 elevation ($3^{\circ }$) for $1^{\circ }\times 1^{\circ }$ grid
Azimuth angles per point	8 ($0^{\circ }:45^{\circ }:315^{\circ }$)

where $A_0$ represents the mean value, $A_1$ and $B_1$ the annual amplitudes, $A_2$ and $B_2$ the semiannual amplitudes of $G_{\mathrm{n_h}}$ and *doy* the day of year. Again, least-squares adjustments are applied in order to fit $A_0$, $A_1$, $B_1$, $A_2$ and $B_2$ to the discrete gradients at each point of the grid. Users can eventually determine the actual gradients for the exact time and location of their measurement through bilinear interpolation from the surrounding grid points. These empirical horizontal gradients are part of the new empirical troposphere model Global Pressure and Temperature 3 (GPT3) (Landskron and Böhm 2017), optionally in $1^{\circ }\times 1^{\circ }$ and $5^{\circ }\times 5^{\circ }$ resolution. Figure 3 shows mean values, cosine amplitudes and standard deviation of $G_{\mathrm{n_h}}$, while Fig. 4 involves the same without the cosine amplitudes for $G_{\mathrm{e_h}}$, $G_{\mathrm{n_w}}$ and $G_{\mathrm{e_w}}$. In the top left plot of Fig. 3, the systematic effect of the atmospheric bulge is predominant. The hydrostatic part generally affects regions outside the tropics, while the wet part is most distinct roughly between 25$^{\circ }$N and 25$^{\circ }$S (center left plot of Fig. 4). In the top left plot of Fig. 4, the systematic effect of the deflections of the vertical can be seen, which are very distinct near dominant mountain ranges such as the Andes or at plate boundaries such as around Japan. The wet gradients (center left and bottom left plots of Fig. 4) are mainly affected by trade winds.

**Fig. 3:**
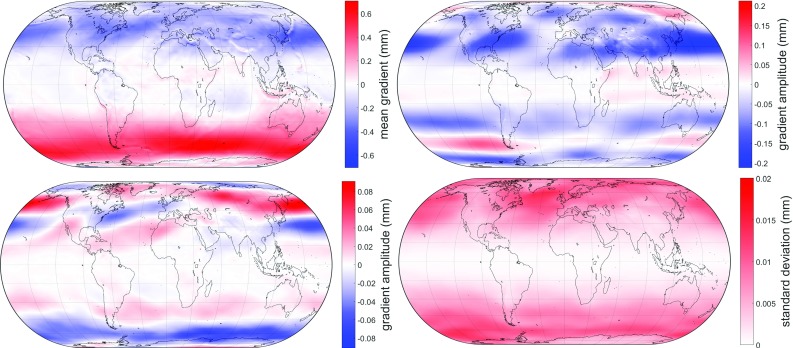
Mean values $A_0$ (top left), annual amplitudes $A_1$ (top right), semiannual amplitudes $A_2$ (bottom left) and standard deviation of the residuals (bottom right) of the hydrostatic north gradient $G_{\mathrm{n_h}}$ from GPT3. $B_1$ and $B_2$ are not included, as they are very similar to $A_1$ and $A_2$

**Fig. 4:**
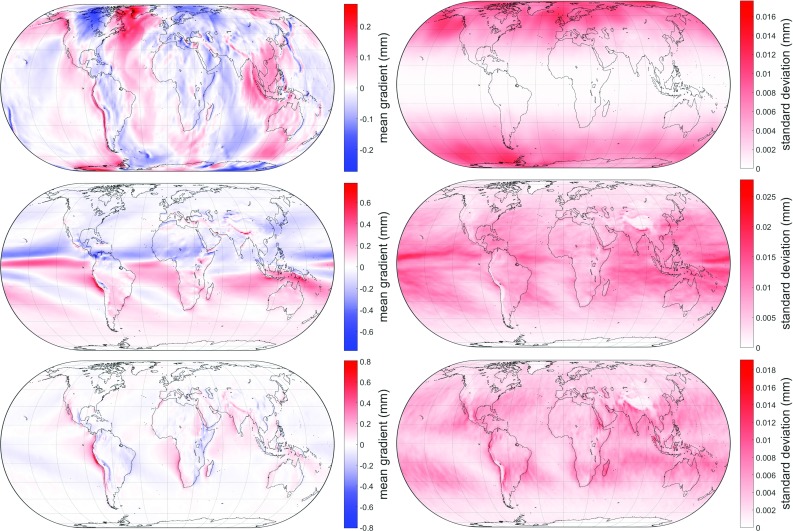
Mean values $A_0$ (left) and standard deviations of the residuals (right) of the hydrostatic east gradient $G_{\mathrm{e_h}}$ (top), wet north gradient $G_{\mathrm{n_w}}$ (center) and wet east gradient $G_{\mathrm{e_w}}$ (bottom) from GPT3. Due to lack of additional information, the amplitudes $A_1$, $B_1$, $A_2$ and $B_2$ are not included here

**Fig. 5:**
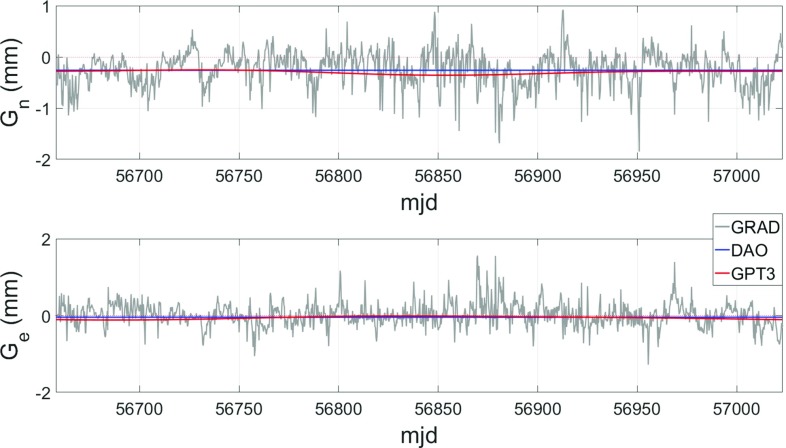
Comparison between total discrete and empirical north (top) and east (bottom) gradients for station WETTZELL during spring 2014. In case of GPT3, the gradients for a specific site are determined through bilinear interpolation from the four surrounding grid points

Empirical gradients, however, only have the ability to describe a small, apparently insignificant part of the actual, discrete gradients, which is outlined in Fig. 5. Unlike DAO, the GPT3 gradients possess a small time-dependent component, although there is no chance to sufficiently describe the significant random, short-term variations due to weather events dominating the behavior of the discrete gradients.

## Comparisons and results

In order to assess the quality of GRAD and GPT3, several comparisons are undertaken. First, BLRs are determined from VLBI analyses using VieVS, as shown in Sect. 4.1. Nine years of VLBI data including 1338 observation sessions are analyzed for this purpose, where only sessions with at least 3 observing stations were picked, eliminating all intensive sessions. Secondly, the gradients are used to model delays which are then compared to ray-traced delays (Sect. 4.2). The better the gradients approximate the ray-traced delays, the higher their accuracy is assumed to be. These comparisons are done on a global grid with a horizontal resolution of $5^{\circ }\times 5^{\circ }$.

**Table 3:** Setting for the VLBI analysis using VieVS

Option	Decision
Mapping function	Vienna Mapping Functions 1 (VMF1) (Böhm et al. 2006)
Terrestrial reference frame	VieVS TRF (Böhm et al. 2017)
Celestial reference frame	International Celestial Reference Frame 2 (ICRF2)
Tidal ocean loading	FES2004 (Lyard et al. 2006)
Tidal and non-tidal atmosphere loading	VIENNA (Wijaya et al. 2013)
Estimate $\varDelta L_w^z$ within the analysis	Yes; as piece-wise linear offsets hourly using relative constraints of 1.5 cm
Estimate gradients within the analysis	if desired; as piece-wise linear offsets 6-hourly using relative constraints of 0.5 mm, but no absolute constraints

**Table 4:** Mean BLRs (cm) from VLBI analyses for all 1338 sessions from 2006 to 2014. In column (1), only a priori gradients are used, while in column (2) the gradients are additionally estimated in the VLBI analysis using the standard gradient formula

Gradient model	(1)	(2)
(a) No a priori gradients	1.68	1.65
(b) LHG	1.66	1.67
(c) GRAD-1	1.58	1.66
(d) GRAD-2	1.57	1.65
(e) GRAD-3	1.58	1.65
(f) APG	1.65	1.66
(g) DAO	1.64	1.66
(h) GPT3 ($5^{\circ }\times 5^{\circ }$)	1.63	1.66
(i) GPT3 ($1^{\circ }\times 1^{\circ }$)	1.63	1.66
Ray-traced delays	1.57	1.64

**Fig. 6:**
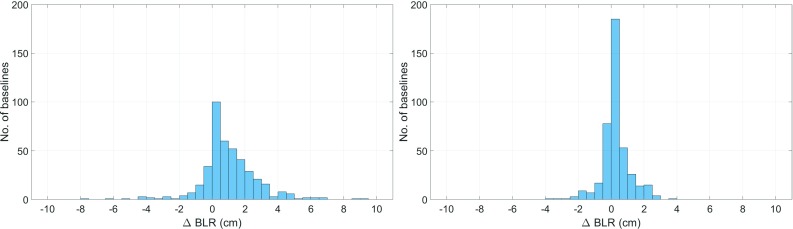
Difference in BLR (cm) from VLBI analysis for all VLBI sessions from 2006 to 2014 without gradient estimation. Left: using GRAD-2 compared to no a priori gradients; right: using GPT3 ($1^{\circ }\times 1^{\circ }$) compared to no a priori gradients. The bars in the positive range imply improvement each

**Fig. 7:**
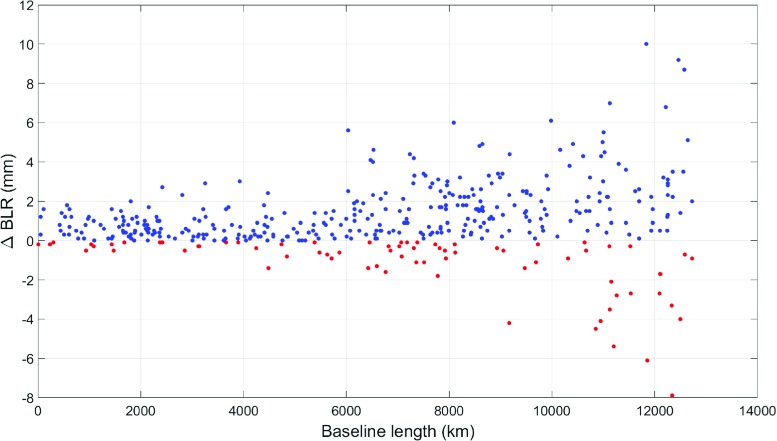
Difference in BLR (cm) from VLBI analysis for all VLBI sessions from 2006 to 2014 without gradient estimation, using GRAD-2 compared to no a priori gradients. Blue dots indicate improvement through using GRAD-2. It can be seen that the improvement is most distinct for shorter baselines

### Comparison of BLRs

Baseline length repeatabilities are an appropriate measure to assess the quality of geodetic VLBI products (Böhm and Schuh 2004; Titov 2009). The lower the BLR, the better the performance of a certain model. Table 4 shows the resulting BLRs from VLBI analyses of several models, as averaged over 1338 VLBI sessions from 2006 to 2014. The ray-traced delays, which serve as the basis for the determination of the GRAD a priori gradients, were computed following the specifications in Table 1. They were then interpolated to the VLBI observation epochs through spline interpolation. The settings for the VLBI analyses are listed in Table 3. The results of Table 4 are surprising because the estimation^1^ of gradients in the VLBI analysis degrades the resulting BLRs. Best results are achieved when using a priori gradients without estimation of the gradients. GRAD-2 yields the best performance, improving the BLRs of 43% of the stations by more than 1 mm while degrading only 5% of the stations by more than 1 mm (the complementary 52% are between − 1 and + 1 mm, too small to be referred to as an improvement or degradation). Figure 6 outlines this more closely, assuming no gradients were estimated in the VLBI analysis. Figure 7 shows that the improvement from the a priori gradients is most distinct for shorter baselines. This is most likely because horizontal gradients affect horizontal positions in particular. Since baselines run straight through the Earth, their repeatability is less affected by horizontal position changes with increasing baseline length.

There is a stark contrast to the results from Landskron et al. (2015b), who concluded that estimating gradients yields best results in any case. The essential difference is that Landskron et al. (2015b) analyzed only two weeks of VLBI data, more precisely the CONT11 campaign. Each session of CONT11 consists of a vast number of observed baselines, in fact more than 4000, providing an optimal basis for the gradient estimation. As a consequence, the lowest BLRs are achieved with the estimation. Although the results of Table 4 contain such sessions as well, the vast majority of sessions comprises only a few hundreds of observations. This substantially impairs the quality of the estimated gradients and is finally reflected in moderate BLRs.

**Table 5:** Mean BLRs (cm) from VLBI analyses for all those sessions from 2006 to 2014 that contain fewer than 3000 observations (1129 out of 1338 sessions, columns 2 and 3) and more than 3000 observations (209 out of 1338 sessions, columns 4 and 5). In (1), only a priori gradients are used, while in (2) the gradients are additionally estimated in the VLBI analysis

Gradient model	< 3000 observations		> 3000 observations	
	(1)	(2)	(1)	(2)
(a) No a priori gradients	2.32	2.38	1.08	0.97
(b) LHG	2.32	2.36	1.06	1.04
(c) GRAD-1	2.21	2.39	1.00	0.99
(d) GRAD-2	2.19	2.37	1.01	0.98
(e) GRAD-3	2.19	2.38	1.01	0.99
(f) APG	2.25	2.39	1.09	0.97
(g) DAO	2.23	2.39	1.09	0.97
(h) GPT3 ($5^{\circ }\times 5^{\circ }$)	2.23	2.39	1.08	0.97
(i) GPT3 ($1^{\circ }\times 1^{\circ }$)	2.23	2.39	1.07	0.97
Ray-traced delays	2.18	2.33	1.01	0.99

Apparently, a session must have a minimum number of observations in order to get reliable results. To prove this assumption, various tests were made which yielded an appropriate boundary value of 3000 observations per session, below which no gradient estimation shall be done.^2^ VLBI analyses carried out separately for all VLBI sessions containing fewer than 3000 observations and for those containing more than 3000 observations result in Table 5. This unambiguously proves the assumption that gradient estimation using a least-squares adjustment shall only be done for sessions possessing a sufficient number of observations. Above 3000 observations per session the gradients shall be estimated, whereas below this boundary it is strongly recommended to not estimate them as the least-squares adjustment will most likely not output well-fitting gradients. This is new as the commonly accepted opinion in VLBI analysis has been to always estimate the gradients for every session. Among the a priori gradients, GRAD-2 performs best, independent from the number of observations. When having fewer than 3000 observations per session, GRAD-2 improves 44% of the BLRs by more than 1 mm while it degrades only 4% by more than 1 mm with respect to no a priori gradients. On the other hand, when having more than 3000 observations per session, GRAD-2 improves 41% of the BLRs by more than 1 mm while it degrades only 9% by more than 1 mm with respect to no a priori gradients. Also the empirical gradient model yields thorough results, particularly for a lower number of observations. When having fewer than 3000 observations per session, GPT3 ($1^{\circ }\times 1^{\circ }$) improves 17% of the BLRs by more than 1 mm while it degrades only 3% by more than 1 mm with respect to no a priori gradients. On the other hand, when having more than 3000 observations per session, GPT3 ($1^{\circ }\times 1^{\circ }$) improves 11% of the BLRs by more than 1 mm while it degrades 7% by more than 1 mm with respect to no a priori gradients. A further pleasant outcome of Tables 4 and 5 is that the results from GRAD-2 are as good as those from the ray-traced delays, indicating that the approximation of the ray-traced delays using the extended gradient formula Eq. (5) works properly. The boundary of 3000 observations might appear a little general, as it does not consider the number of stations participating in a session or any geometry in the station constellation; however, it turned out to be very appropriate and useful. Alternatively, it would also be possible to apply tight absolute constraints to the gradients for sessions with a low number of observations instead of the a priori gradients. This, however, was not tested in this investigation.

**Fig. 8:**
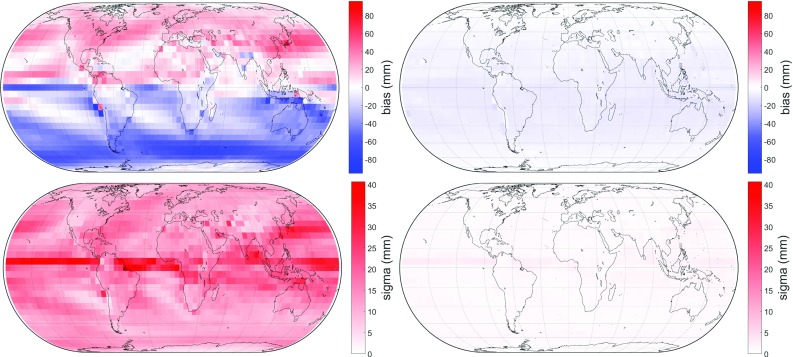
Bias (top) and standard deviation (bottom) of the residuals in slant total delay at $5^{\circ }$ elevation and 180$^{\circ }$ azimuth. Top left: bias of residuals between ray-tracing and disregarding azimuthal symmetry. Due to the atmospheric bulge, the residuals are generally positive in the northern hemisphere and generally negative in the southern hemisphere. Top right: bias of residuals between ray-tracing and GRAD-1; the residuals are considerably lowered, albeit slightly negative. Bottom left and bottom right: the respective standard deviations. The application of GRAD-1 tremendously reduces the residuals at all levels as well

The following itemization sums up all facts concerning the BLR analysis.

- The ray-traced delays, which represent the absolute reference values in this comparison, can be approximated perfectly well by using VMF1 plus the gradients GRAD-1 as well as GRAD-2. In other words, this means that better BLRs can only be attained as soon as the ray-traced delays themselves become more accurate.
- Unlike the commonly accepted opinion, gradients shall not always be estimated within VLBI analysis. The design matrix in the least-squares adjustment must be sufficiently over-determined in order to produce reliable results. A certain criterion has to be fulfilled to ensure this, where the minimum value of 3000 observations per session turned out to be an approximate, but reliable boundary. Below this number, no gradients shall be estimated in VLBI analysis.
- Best results are achieved with the a priori gradients GRAD-2. However, GRAD-1 is only marginally worse but does not require a new gradient formula.
- Empirical a priori gradients generally have a considerably smaller effect on the resulting BLRs than discrete a priori gradients. In case no discrete a priori gradients are available, empirical gradients are most useful for VLBI sessions with few observations, where its usage yields much better BLRs than estimating the gradients in the analysis. GPT3 is marginally better that APG and DAO, whereas the difference between GPT3 ($5^{\circ }\times 5^{\circ }$) and GPT3 ($1^{\circ }\times 1^{\circ }$) is even more marginal.
- The topography has a significant influence on the resulting gradients, e.g., the presence of mountain ranges causes variant gradient values. For this reason, the provision on a grid with a coarse mesh size of $5^{\circ }$ seems to be insufficient, as the grid points are up to 550 km away from each other that makes it impossible to consider any topography in between. The mesh size of $1^{\circ }$ comes closer to reality in theory; however, the results are only slightly better. Probably, the provision of new empirical gradients for individual sites would yield better results for VLBI purposes than on a global grid. The provision on a global grid, however, allows GPT3 to be used for many more purposes than VLBI.
- In general, GRAD provides better BLRs than the LHG from Böhm and Schuh (2007).
- When deciding to estimate gradients, the use of a priori gradients only slightly affects resulting BLRs.

### Comparison of modeled delays with ray-traced delays

Unlike the comparison in section 4.1 where gradients were determined for VLBI stations located at discrete spots on Earth, in this section a comparison is done for a $5^{\circ }\times 5^{\circ }$ global grid containing 2592 grid points. Ray-traced delays were generated for each grid point according to the specifications listed in Table 2. The ray-traced delays, regarded as the "true" values, are then compared to delays modeled with the three gradient formulae Eqs. (2), (5) and (6). The gradients LHG as well as DAO cannot be considered here, as they are only available for VLBI station locations and not for arbitrary points such as grid intersections.

**Fig. 9:**
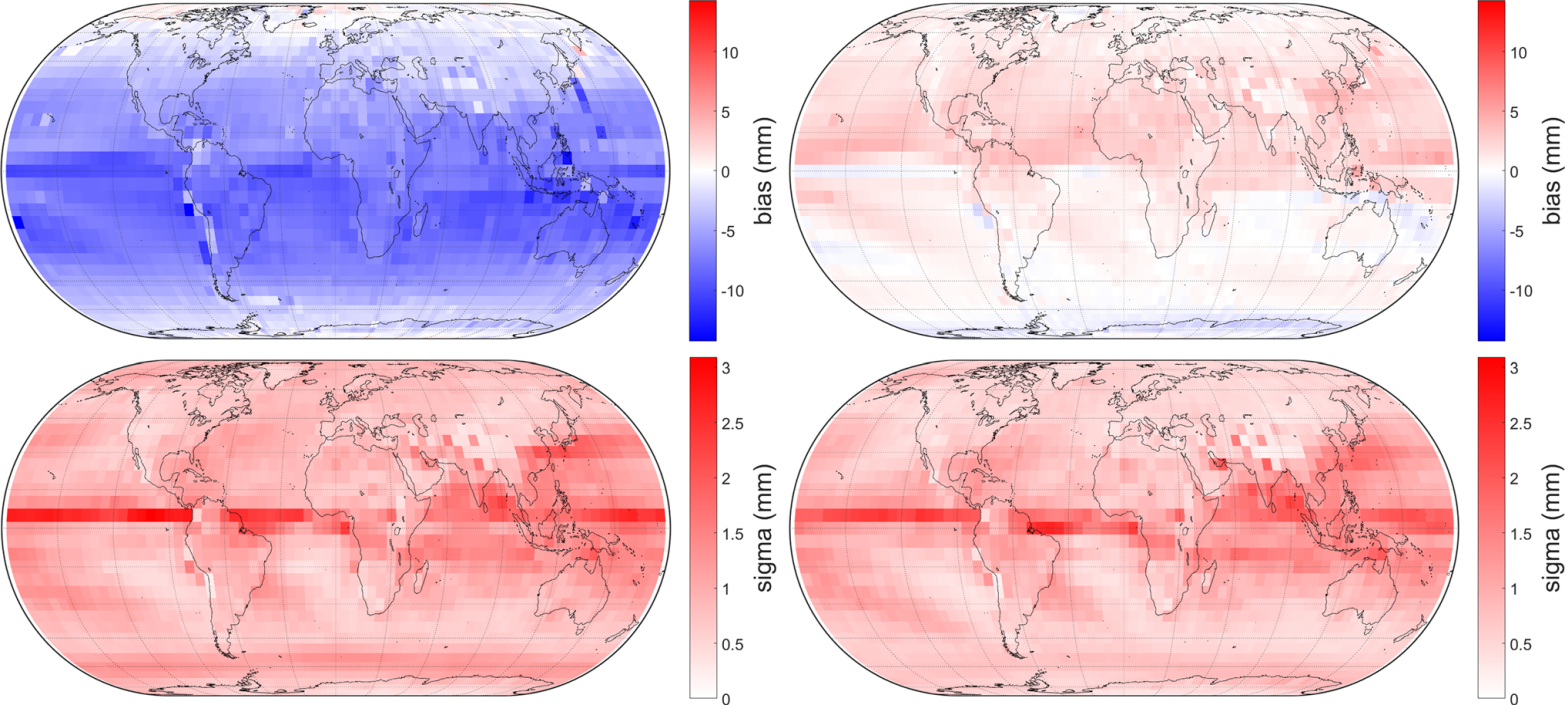
Bias (top) and standard deviation (bottom) of the residuals in slant total delay at 5$^{\circ }$ elevation and 180$^{\circ }$ azimuth. Top left: bias of residuals between ray-tracing and applying GRAD-1; this is equal to the top right plot of Fig. 8, but differently scaled. Top right: bias of residuals between ray-tracing and GRAD-2. Thus, the negative residuals are mainly removed. Bottom left and bottom right: the respective standard deviations, showing no noticeable difference between them

This comparison is made concerning the residuals between the azimuth-wise ray-traced delays and those averaged over all azimuths for each of the 2592 grid points, 120 epochs, 8 azimuths and 4 elevation angles. GRAD gradients are progressively applied in order to reveal their performance in reducing the residuals between the modeled delays and the ray-traced delays. Figures 8 and 9 feature the improvement of the residuals in bias and standard deviation, respectively, resulting from the application of GRAD.

Comparing mean absolute residuals or mean absolute error (MAE) is very meaningful, too. It describes the total difference to the reference values averaged over all observations, whereas the bias is always dependent on the algebraic sign. Table 6 lists mean absolute residuals for the different GRAD gradients averaged over all grid points and epochs, sorted by azimuth. The Vienna Mapping Functions 3 (VMF3) (Landskron and Böhm 2017) is used for modeling the azimuthally symmetric part of the delay. It does not matter here which GPT3 version to use, as the comparison is done for the $5^{\circ }\times 5^{\circ }$ grid intersection points.

**Table 6:** Mean absolute residuals (mm) in slant total delay between ray-tracing and applying no gradient formula, the three GRAD gradients and empirical gradients, each for 5$^{\circ }$ elevation and different azimuths $\alpha$, averaged over all 2592 grid points and 120 epochs from January 2001 to December 2010

Gradient model	Mean abs. diff. in $\varDelta L$ (cm)					
	$\alpha = 0^{\circ }$	$\alpha = 45^{\circ }$	$\alpha = 90^{\circ }$	$\alpha = 135^{\circ }$	$\alpha = 180^{\circ }$	Mean $\alpha$
No a priori gradients	25.6	19.6	9.7	19.0	26.0	20.0
GRAD-1	4.1	1.1	4.1	1.1	4.2	2.9
GRAD-2	1.4	0.8	1.1	0.8	1.3	1.1
GRAD-3	1.4	0.8	1.1	0.8	1.3	1.1
APG	16.4	14.4	10.8	13.0	16.8	14.3
GPT3	9.4	7.5	7.4	7.5	9.5	8.3

From Table 6 the following conclusions can be drawn:

- Due to the presence of an atmospheric bulge, azimuthal asymmetry is most pronounced in north and south direction and is least pronounced in east and west direction.
- The consideration of azimuthal asymmetry is of particular importance especially for small elevation angles like $5^{\circ }$.
- With the standard gradient formula of Chen and Herring (1997) ($=$ GRAD-1), an improvement in the slant total delays of up to 20 mm can be reached at 5$^{\circ }$ elevation. On average, it improves the residuals by 86%.
- Using the second gradient formula ($=$ GRAD-2) further improves the slant total delays, although to a smaller degree. On average, the residuals are lower by notable 95% compared to not considering azimuthal asymmetry.
- The third gradient formula ($=$ GRAD-3) is not meaningful as it is not capable of further reducing the residuals compared to GRAD-2. This is most likely owing to insufficient over-determination in the least-squares adjustment, where six gradient variables shall be estimated from eight azimuths.
- The residuals when using empirical gradients are far off those from discrete gradients. However, GPT3 considerably improves the delays with respect to APG.

## Conclusions

On the basis of ray-traced delays through numerical weather models (NWMs) using the highly sophisticated VieVS ray-tracer (Hofmeister and Böhm 2017), we developed new discrete horizontal gradients for a priori use in VLBI analysis referred to as GRAD, as well as a new empirical gradient model GPT3 in the two grid sizes $1^{\circ }\times 1^{\circ }$ and $5^{\circ }\times 5^{\circ }$. All of these models are capable of outperforming existing models in our comparisons; this is shown through baseline length repeatabilities (BLRs) from VLBI analyses as well as theoretical delays. An extended gradient formula including higher-order terms (GRAD-2) is able to simulate the ray-traced delays with even higher precision than the standard gradient formula by Chen and Herring (1997). We found that the common estimation of gradients in VLBI analysis shall only be carried out under certain conditions. If the respective VLBI session exhibits fewer than 3000 observations, the gradient estimation rather degrades than improves the results. The sole usage of a priori gradients GRAD without additional gradient estimation is to be preferable in 90% of the VLBI sessions. However, as in general only a comparably small improvement can be achieved with the new models, we are forced to the conclusion that a big leap in the accuracy may only be achieved when the ray-traced delays and NWMs themselves become more accurate. This is supported by the fact that the ray-traced delays can be approximated already very well through the modeled gradients in all comparisons.

## Data and code availability

Text files containing GRAD gradients can be downloaded from http://ggosatm.hg.tuwien.ac.at/DELAY/ETC/GRAD/. Information on the usage of the files is found in http://ggosatm.hg.tuwien.ac.at/DELAY/readme.txt.

## Footnotes

1 The estimation of gradients is done with the standard gradient formula Eq. (2); an estimation using the extended gradient formulae Eqs. (5) and (6) was tested as well, but turned out to not improve the results.

2 There were several strategies tested for a boundary value regarding total number of observations, mean number of observations per station, number of scans and some more. However, the most general criterion (total number of observations) turned out to be the most appropriate one at the same time. See Landskron (2017) for details.
